# Validity and reliability of the Patient Health Questionnaire-8 in Swedish for individuals with systemic sclerosis

**DOI:** 10.1007/s00296-020-04641-1

**Published:** 2020-07-13

**Authors:** Malin Mattsson, Gunnel Sandqvist, Roger Hesselstrand, Annica Nordin, Carina Boström

**Affiliations:** 1 Department of Neurobiology, Care Sciences and Society, Karolinska Institutet, Huddinge, Stockholm, Sweden; 2 Department of Physiotherapy, Sunderby Hospital, Luleå, Sweden; 3 Department of Clinical Sciences, Lund University, Lund, Sweden; 4 Department of Rheumatology, Skåne University Hospital, Lund, Sweden; 5 Department of Medicine Solna, Karolinska Institutet, Stockholm, Sweden; 6 Unit of Rheumatology, Karolinska University Hospital, Stockholm, Sweden; 7 Allied Health Professionals Function, Karolinska University Hospital, Stockholm, Sweden

**Keywords:** Depression, Scleroderma systemic, Patient-reported outcome measures, Psychometrics, Quality of life

## Abstract

**Background:**

Depressive symptoms are common in rheumatic diseases and influence patients’ quality of life. The Patient Health Questionnaire-9 (PHQ-9), which assesses symptoms of depression, is valid in English in patients with systemic sclerosis (SSc). However, the measurement properties of the PHQ-8 (short version of the PHQ-9) have not been evaluated in Swedish patients with SSc.

**Objective:**

To investigate different aspects of validity and reliability of the PHQ-8 in Swedish (PHQ-8 Swe) for individuals with SSc.

**Methods:**

A total of 101 patients with SSc participated. Content validity was evaluated via interviews of 11 patients and 10 health professionals. Construct validity, internal consistency test–retest reliability, and floor/ceiling effects were evaluated in 90 patients.

**Results:**

Content validity was satisfactory, but some linguistic adjustments were made. Confirmatory factor analysis supported a better fit for a two-factor structure. Moderate-to-strong correlations were found between the PHQ-8 Swe and scleroderma HAQ including VAS (*r*_s_ = 0.4–0.7); Multidimensional Assessment of Fatigue (*r*_s_ = 0.7); RAND-36 subscales (*r*_s_ = − 0.5 to − 0.8); and lung disease severity (Medsger scores) (*r*_s_ = 0.4). There were weak correlations (*r*_s_ = <0.4) between the PHQ-8 Swe and modified Rodnan skin score; and vascular, heart, and kidney disease severity. Cronbach’s alpha was 0.85, corrected item-to-total correlations were >0.40, and the ICC for the total score was 0.83. No floor/ceiling effects were found.

**Conclusion:**

The PHQ-8 Swe has satisfactory content validity and sufficient reliability in patients with in majority limited SSc. It is more strongly associated with self-reported disability, pain, disease interferences with daily activities, fatigue, and quality of life than with disease severity, except for a moderate association with lung severity.

## Introduction

Systemic sclerosis (SSc), or scleroderma, is a multisystem autoimmune inflammatory disorder. The disease is characterized by microvascular damage and increased deposition of collagen and other matrix molecules in skin and organ systems [1]. SSc is more prevalent among women, with a female-to-male ratio of 4.7:1 [2]. The clinical course can vary from limited skin thickening to severe organ damage such as pulmonary fibrosis or pulmonary arterial hypertension. The disease can be divided into two subtypes depending on the extent of fibrotic skin involvement: limited cutaneous SSc (lcSSc) and diffuse cutaneous SSc (dcSSc) [1]. There is currently no cure for SSc; thus, treatments focus on reducing disease manifestations and improving health-related quality of life (HRQL) [3].

Depression is more common in SSc patients than in patients with other rheumatic diseases [4, 5]. Symptoms of depression occur in approximately one-third to two-thirds of patients with SSc [6], depending on which questionnaire is used and whether or not the prevalence of depression is based on valid interview methods [7]. Disease-specific symptoms such as reflux, constipation, dyspnea, digital ulcers, pain, fatigue, and changes in physical appearance are associated with negative emotions in SSc [4]. Depressive symptoms are associated with poor HRQL [8]. Patients with SSc who have depressive symptoms are also reported to be less physically active than those without depressive symptoms [9]. Further, depressive symptoms are associated with lower self-efficacy and reduced likelihood of adopting health-promoting behaviors [6, 10].

Depressive symptoms are important to detect and address in patients with SSc. One possible way to capture symptoms of depression is to use patient-reported outcome measures (PROMs) [4]. The Patient Health Questionnaire-9 (PHQ-9) is one such PROM that is reliable and valid in SSc in for example English [11, 12]. The basis of the items in the PHQ-9 is also equivalent to the criteria for depression [13]. A slightly shorter version, the PHQ-8, also exists, wherein the ninth item (thoughts of self-harm and death) is omitted [13]. A high correlation between the PHQ-9 and the PHQ-8 has been found in patients with SSc, and the PHQ-8 is preferred in SSc [14]. Thus, a Swedish version of the PHQ-8 for patients with SSc was of interest for the present study. Health professionals may use the PHQ-8 to detect and facilitate communication about symptoms of depression [15–17], support self-management of these symptoms, and refer patients to the appropriate healthcare provider.

The PHQ-8 in Swedish has not been psychometrically evaluated in SSc. However, a linguistic validated version of the PHQ-9 in Swedish can be found on the Pfizer website [18]. A Swedish version of the PHQ-9 has support for internal consistency and concurrent validity among patients with affective disorder diagnoses; meanwhile, high internal consistency and structural validity among patients with self-reported depression have been reported [19, 20]. To determine the quality of PROMs, their measurement properties in the target population need to be studied and the ability of patients to properly understand questions about symptoms of depression is of importance [21]. The present study aimed to investigate different aspects of the validity and reliability of the PHQ-8 in Swedish for individuals with SSc.

## Methods

This psychometric study included content validity, construct validity (structural validity and hypotheses testing), internal consistency, test–retest reliability, and floor and ceiling effects [21].

### Participants

Study participants were recruited from three rheumatology centers in Sweden. The inclusion was based on: diagnosis meeting the 2013 ACR/EULAR criteria for SSc [22], being ≥18 years of age, disease duration of ≥1 year, and ability to understand and speak Swedish. To evaluate content validity, 11 patients from one center agreed to participate, and fulfilled the inclusion criteria (Table 1), while 10 health professionals (HPs) with various occupational backgrounds from two centers were invited and agreed to participate (Table 2). To evaluate construct validity, internal consistency, test–retest reliability, and floor and ceiling effects, 90 patients who fulfilled the criteria for the study from two centers (*n* = 35, *n* = 55, respectively) consented to participate (Tables 1, 3). According to the consensus-based standards for the selection of health status measurement instruments (COSMIN) checklist, a sample size of ≥7 individuals (patients or HPs) is considered to be very good for purposes of assessing content validity by qualitative method [21]. Further, a sample size of 50–99 individuals is found to be adequate for the employed assessment of construct validity, internal consistency, and test–retest reliability [21].

**Table 1:** Characteristics of patients with systemic sclerosis (SSc)

	Content validity (*n* = 11)^a^	Construct validity, reliability, and floor and ceiling effects (*n* = 90)^C^
*Sociodemographic data*		
Women, *n* (%)	10 (91)	76 (84)
Age in years, median (IQR)	60 (48–68)	61 (50–70)
Civil status, *n* (%)		
Married or living together	8 (73)	65 (72)
Single	3 (27)	25 (28)
Level of education, *n* (%)		
College or university	5 (45)	48 (55)
High school	5 (45)	28 (32)
Vocational school or other secondary school	1 (9)	3 (3)
Elementary school	0 (0)	9 (10)
Professional status, *n* (%)		
Employed, full- or part-time	6 (56)	35 (39)
Student or unemployed	0 (0)	1 (1)
Sick-listed, full- or part-time	1 (9)	13 (14)
Retired early, full- or part-time	3 (27)	11 (12)
Retired	3 (27)	37 (41)
*Disease variables*		
Disease duration in years^b^, median (IQR)	11 (6–18)	8 (4.0–14.3)
Limited/diffuse cutaneous SSc, *n* (%)	9 (82)/2 (18)	70 (78)/20 (22)
mRSS score 0–51, median (IQR)	14 (6–26)	2 (0.8–4.0)
MSS score 0–4, median (IQR): 0–1, 2–4^c^, *n* (%)		
Peripheral vascular system	1 (1–2): 6 (55), 5 (45)	1 (1–1.3): 68 (76), 22 (24)
Lung system	2 (2–3): 2 (18), 9 (82)	1 (0–2): 61 (68), 29 (32)
Heart system^D^	0 (0–1): 10 (100), 0 (0)	0 (0–0): 84 (93), 6 (7)
Kidney system	0 (0–0): 11 (100), 0 (0)	0 (0–0): 88 (99), 1 (1)
Other rheumatic disease^A, d^, *n* (%)		24 (27)
Comorbidity, *n* (%)		
Cardiovascular disease^B^	1 (9)	19 (21)
Thromboembolism^B^	1 (9)	3 (3)
Cancer	0 (0)	6 (7)
Diabetes	0 (0)	1 (1)
Depression or other psychological disorders	0 (0)	12 (13)
Treatment, *n* (%)		
Proton pump inhibitors	8 (73)	72 (81)
Calcium channel blockers	7 (63)	66 (74)
Immunosuppressive treatment	3 (27)	36 (40)
Corticosteroids, *n* (%)*,* mean (SD)	2 (18) 2.5 mg/5 mg	18 (20), 4.6 mg (SD 2.3)
NSAID, paracetamol, opioids	0 (0), 7 (34), 0 (0)	18 (20), 35 (39), 10 (11)
Antidepressants, anxiolytics	0 (0), 0 (0)	9 (10), 3 (3)

*IQR* interquartile range; *SD* standard deviation

*mRSS* modified Rodnan skin score, *MSS* Medsger Severity Scale

^A^Other rheumatic diseases in at least two patients

^B^Patients could have one or two diseases/conditions

^C^1 or 2 missing values for certain variables

^D^1 missing value in the *n* = 11 sample

^a^Not included in the test of construct validity, reliability, and floor and ceiling effects

^b^Disease duration referred to the time from the first non-Raynaud’s symptom

^c^*Score interval 0–1 *normal to mild, *score interval 2–4* moderate to end-stage

^d^Sjögrens syndrome (*n* = 13), myositis (*n* = 7), systemic lupus erythematosus (*n* = 2), rheumatoid arthritis (*n* = 2)

**Table 2:** Characteristics of health professionals (*n* = 10)

	Content validity
Woman, *n* (%)	8 (80)
Age in years, median (IQR)	55.5 (42.3–63)
Years in the profession, median (IQR)	21 (12.3–32.3)
Years working in rheumatology, median (IQR)	11 (4.8–19.5)
Years working with patients with SSc, median (IQR)	5.5 (3.5–15.0)
Profession	*n*
Nurse Occupational therapist Physicians Physiotherapist Social worker	2 2 2 2 2

*IQR* interquartile range, *SSc* systemic sclerosis

**Table 3:** Scores of the patient-reported outcome measures used to assess construct validity (*n* = 90)

Patient-reported outcome measures^A^	
SHAQ score 0–3, median (IQR)	
HAQ-DI	0.38 (0.13–0.88)
HAQ-DI VAS^a^	
Pain	0.74 (0.10–1.50)
SHAQ VAS^a^	
Gastrointestinal symptoms	0.18 (0.02–1.08)
Lung symptoms	0.14 (0.02–0.98)
Raynaud’s phenomenon	0.68 (0.12–1.46)
Digital ulcers	0.04 (0.00–0.40)
Overall disease severity	0.77 (0.20–1.47)
MAF score 1–50, median (IQR)	25.4 (16.4–34.6)
RAND-36 score 0–100, median (IQR)	
Physical function	67 (50–85)
Physical role function	25 (0–100)
Bodily pain	68 (45–90)
General health	45 (30–60)
Vitality	55 (40–75)
Social function	75 (63–100)
Emotional role function	100 (33–100)
Mental health	80 (64–92)

*IQR* interquartile range, *SHAQ* Scleroderma Health Assessment Questionnaire, *HAQ-DI* Health Assessment Questionnaire-Disability Index, *VAS* visual analogue scale, *MAF* Multidimensional Assessment of Fatigue, *RAND-36* RAND 36-Item Health Survey

^A^1–6 missing values occurred in certain patient-reported outcome measures

^a^VAS is 15 cm; VAS score was multiplied by 0.2 to attain a score of 0–3

### Disease severity variables

Disease severity variables were collected by a rheumatologist. The modified Rodnan skin score (mRSS) evaluates skin involvement. Skin thickness is scored by palpation of the skin in 17 body areas, each of which is scored from 0 (uninvolved) to 3 (severe thickening). The mRSS is reliable and valid in SSc [23].

The Medsger severity scale (MSS) assesses the severity of the disease in nine organ systems. Each organ system is scored separately according to the following: 0 (normal), 1 (mild), 2 (moderate), 3 (severe), and 4 (end-stage). Aspects of validity have been confirmed for MSS in SSc [23]. The following organ systems were used in our study: peripheral vascular, lung, heart, and kidney.

### Patient-reported outcome measures

Patients completed PROMs. The PHQ-8 assesses the frequency of depressive symptoms over the past two weeks (Table 4). The items’ response options are scored from 0 to 3 and are summed to generate a total score with a range of 0–24. A score interval of 0–4 indicates no significant depressive symptoms, 5–9 indicates mild depressive symptoms, 10–14 is moderate, 15–19 is moderately severe, and 20–24 is severe [13, 24]. A final item is not included in the total score and is addressed to patients who indicated any problems among the responses; it asks how difficult these problems have made it for the patients to function in different daily life situations. In our study, the response options in that question were scored as follows: not difficult at all = 0, somewhat difficult = 1, very difficult = 2, and extremely difficult = 3. This final item was used in our study to assess if patients had changed during the test–retest period. The PHQ-8 was completed as self-report by paper and pencil.

**Table 4:** Test–retest reliability of the Patient Health Questionnaire-8 in Swedish for individuals with systemic sclerosis (*n* = 90)

Over the last 2 weeks, how often have you been bothered by any of the following problems? Use an “ ” to indicate your answer *Hur ofta har du besvärats av något/några av följande problem de senaste 2 veckorna?* *Kryssa i lämpligt svarsalternativ per fråga* Response options Not at all = 0; several days = 1; more than half the days = 2; nearly every day = 3 *Inte alls; Flera dagar; Mer än hälften av dagarna; Nästan varje dag*	Test Median (IQR) (*n* = 89–90)	Retest Median (IQR) (*n* = 79–81)	Weighted kappa coefficient (*n* = 79–81)	Sign test (*n* = 79–81)
1. Little interest or pleasure in doing things *Känt mindre intresse eller glädje av att göra saker*	1 (0–1) (*n* = 90)	1 (0–1) (*n* = 79)	0.62	1.00
2. Feeling down, depressed, or hopeless *Känt dig nedstämd, deprimerad eller upplevt en känsla av hopplöshet*	0 (0–1) (*n* = 89)	0 (0–1) (*n* = 81)	0.70	0.63
3. Trouble falling or staying asleep, or sleeping too much *Haft svårigheter att somna eller få en sammanhängande sömn eller sovit för mycket*	1 (0–2) (*n* = 89)	1 (0–1) (*n* = 81)	0.74	0.002
4. Feeling tired or having little energy *Känt dig trött eller haft för lite energi*	1 (1–2) (*n* = 89)	1 (1–2) (*n* = 80)	0.60	0.46
5. Poor appetite or overeating *Haft dålig aptit eller ätit för mycket*	0 (0–1) (*n* = 90)	0 (0–1) (*n* = 81)	0.79	0.45
6. Feeling bad about yourself—or that you are a failure or have let yourself or your family down *Tyckt illa om dig själv eller känt dig misslyckad eller att du svikit dig själv eller din familj*	0 (0–1) (*n* = 89)	0 (0–1) (*n* = 81)	0.75	1.00
7. Trouble concentrating on things, such as reading the newspaper or watching television *Haft svårigheter att koncentrera dig på saker, till exempel att läsa tidningen eller se på TV*	0 (0–1) (*n* = 90)	0 (0–1) (*n* = 81)	0.76	0.42
8. Moving or speaking so slowly that other people could have noticed? Or the opposite—being so fidgety or restless that you have been moving around a lot more than usual *Rört dig eller talat så långsamt att andra människor kan ha märkt det, eller motsatsen, att du varit så nervös eller rastlös att du rört dig mer än vanligt*	0 (0–0) (*n* = 90)	0 (0–0) (*n* = 81)	0.64	0.42

The English version is kindly permitted from Professor Kurt Kroenke (personal communication, 2018)

*IQR* interquartile range

The Scleroderma Health Assessment Questionnaire (SHAQ) was used to assess disability, pain, and disease interference with daily activities. The SHAQ comprises the HAQ-Disability Index (HAQ-DI) with 20 items covering daily activities that result in a total score ranging from 0 (no disability) to 3 (severe disability) and one visual analogue scale (VAS) to assess pain (0–15 cm). The SHAQ includes five additional VAS items that assess gastrointestinal symptoms, lung symptoms, Raynaud’s phenomenon, digital ulcers, and overall disease severity interference with daily activities [25]. The value of the VAS is multiplied by 0.2 to attain a score of 0 to 3. Aspects of reliability and validity have been established for the Swedish SHAQ among patients with SSc [26].

The Multidimensional Assessment of Fatigue (MAF) assesses fatigue with 16 items resulting in a cumulative score ranging from 1 to 50. Elevated scores indicate greater fatigue. Aspects of reliability and validity of the Swedish MAF have been confirmed in patients with SSc [27].

The RAND 36-Item (RAND-36) Health Survey was used to assess HRQL. The RAND-36 contains 36 items divided into the following subscales: physical function, physical role function, bodily pain, general health, vitality, social function, emotional role function, and mental health. The subscale total scores range from 0 to 100, where a higher score indicates a better HRQL. The RAND-36 is comparable with the Medical Outcomes Study 36-item Short-Form Health Survey (SF-36) [28], which has been validated in SSc [29].

### Procedures

Evaluation of the PHQ-8 in Swedish (PHQ-8 Swe) for individuals with SSc underwent four steps (see the Appendix). (1) The PHQ-8 Swe was developed from an existing Swedish version of the PHQ-9 [18] by omitting item nine. Adjustment of the Swedish translation to individuals with SSc was approved by Professor Kurt Kroenke (personal communication, 2017). (2) A semi-structured interview guide was developed and used for interviews with patients with SSc and HPs within SSc care to evaluate the content validity of the PHQ-8 Swe (Table 5). The interviews were audio-recorded, transcribed verbatim, and analyzed with content analysis. (3) The research team undertook some linguistic adjustments following the analysis of content validity. In addition, two patient research partners reviewed and commented on the PHQ-8 Swe. The PHQ-8 Swe was then back-translated into English for a comparison with the original; no significant changes were found. (4) The PHQ-8 Swe was further evaluated for construct validity, internal consistency, test–retest reliability, and floor and ceiling effects.

**Table 5:** Interview guide to evaluate content validity of the Patient Health Questionnaire-8 in Swedish

What do you think about the comprehensibility of the items?
Are there any items that are difficult to understand?
What do you think about the relevance of the items to what may be experienced in systemic sclerosis (Patients)
Do the questions reflect all relevant aspects of the symptoms of depression in systemic sclerosis? (HPs)
Would you like to include any items?
Would you like to exclude any items?
What do you think about the instruction and the response options?
Overall, how do you experience the questionnaire?
Would you like to add anything regarding the questionnaire?
To elaborate the answers during the interviews, probes were used to obtain further details (e.g., Would you like to explain it further? If so, then why?, etc.)

*HPs* health professions

The vast majority of patients completed PROMs and answered questions about sociodemographic data in conjunction with their visits to the hospital (first occasion in the test–retest procedure). The PHQ-8 Swe was completed a second time at the patient’s home and returned by mail in a pre-stamped envelope (retest occasion). The average time interval between the test and retest occasions was 11 (SD 7.4) days.

### Ethics approval

The regional committee at Umeå University (No. 2017/149-31) approved the study. Informed written consent was obtained from all patients and HPs who participated in the study in accordance with the Helsinki Declaration. All patients had access to a social worker at the clinic. In the case of severe symptoms of depression, they could be referred to either a psychologist, psychiatrist, or general practitioner.

### Statistical analysis

Construct validity by structural validity was analyzed by confirmatory factor analysis (CFA). Single- and two-factor models (cognitive/affective factor items 1, 2, 6, and 7; somatic factor items 3, 4, 5, and 8) were tested based on previous findings of the PHQ-9 in SSc [11]. The maximum likelihood estimation was used to fit the CFA model. Indexes to assess the fitness of the model were: comparative fit index (CFI), root-mean-square error of approximation (RMSEA), and Chi-square/degree of freedom (CMIN/DF). The following cutoff values were used as the level of acceptance with fit considered acceptable when the CFI was ≥0.90 [30]; the RMSEA was ≤0.08 [31], and the CMIN/DF was <3 [32]. The Akaike information criterion (AIC) was used to compare the factor models, and the lowest AIC indicates a more favorable trade-off between fit and complexity [33].

Hypotheses testing for construct validity in terms of different associations between the total score of the PHQ-8 Swe and the other outcome measures; SHAQ (HAQ-DI and VAS scales), MAF, RAND-36, mRSS, and MSS were evaluated. From the previously reported results in SSc of the PHQ-9 [12, 34], for convergent validity [21], we expected that the PHQ-8 Swe would have at least a moderate correlation with SHAQ (HAQ-DI and VAS scales), MAF, and RAND-36. For divergent validity [35], weak correlations were expected with mRSS, MSS, and disease duration. Spearman’s rank correlation coefficient (*r*_s_) was used, as most of our data are ordinal. Correlation interpretations were as follows: 0 = no association; 0.1–0.3 = weak; 0.4–0.6 = moderate; 0.7–0.9 = strong; and 1.0 = perfect [36]. The calculated correlation coefficient values were rounded to one decimal.

Internal consistency was determined with Cronbach’s alpha coefficient; an alpha coefficient of ≥0.70 was suggested to be sufficient [37]. The corrected item-to-total correlation was also analyzed and item correlations of >0.30 were interpreted as good [38]. Test–retest reliability was assessed by having patients with SSc complete the PHQ-8 Swe on two occasions. The sign test was used to evaluate whether any statistically significant differences were found between test occasions for the total score and each item. The total score was assessed using an intraclass correlation coefficient (ICC), in a two-way mixed model, and absolute agreement [39]. An ICC of ≥ 0.70 is considered sufficient when evaluating test–retest reliability [37]. Weighted kappa with quadratic weights was calculated to analyze the agreement between test occasions for each item [40]. Kappa was interpreted as follows: kappa <0.00 = poor; 0.00–0.20 = slight; 0.21–0.40 = fair; 41–0.60 = moderate; 0.61–0.80 = substantial; and 0.81–1.00 = almost perfect [41].

Floor and ceiling effects were defined as >15% of patients obtaining the lowest or highest possible total score [40].

Missing items on the PHQ-8 Swe were handled as follows: No total score was calculated if two items were missing. If one item was missing, the missing score was replaced by the mean of the completed items [13]. Missing items were not replaced when individual items in the PHQ-8 Swe were investigated. Missing items on the other PROMs were treated as described by the developer of the respective PROMs. The choice of statistical tests was supported by the COSMIN checklist [21, 40]. The level of significance was specified at p ≤ 0.05. Statistical analyses were performed using the SPSS 25, CFA was performed using Amos 25, and weighted kappa was calculated by the VassarStats: Website for Statistical Computation.

## Results

Of the total of 101 patients, most had lcSSc and, at the median, mild disease severity in their peripheral vascular system, as well as normal heart and kidney systems (Table 1). The patients (*n* = 90) participating in the testing of aspects of construct validity and reliability had, at the median, mild disease severity of the lung system. The patients (*n* = 11) in the assessment of content validity had, at the median, moderate disease severity of the lung system and, at the median, greater skin involvement than those in the assessment of construct validity and reliability (Table 1).

The PHQ-8 Swe total score was at median 6 (interquartile range (IQR): 2–12; *n* = 11), the PHQ-8 Swe total score for *n* = 90 patients see aspects of reliability. Of the patients (*n* = 90) who completed the PHQ-8 Swe, 53% had no significant depressive symptoms, 30% had mild symptoms, 15% had moderate symptoms, 1% had moderately severe symptoms, and 1% had severe symptoms. The final item in the PHQ-8 (not included in the total score), assessing the difficulties of symptoms of depression in different daily life situations, was, at the median, in the first measurement occasion 1 (i.e., “somewhat difficult”; min–max: 0–3; *n* = 81) and at retest 1 (i.e., “somewhat difficult”; min–max: 0–2; *n* = 72). There were no statistical changes over time (*p* = 0.84).

### Content validity and linguistic adjustments

The results of the evaluation of the content validity of the PHQ-8 Swe are presented in the domains of comprehensibility, relevance, and comprehensiveness [21], with illustrative quotations in Table 6. Overall, the PHQ-8 Swe was experienced as being easy to understand, relevant in item content, and covering important aspects of depression in SSc. However, the following main changes to the PHQ-8 Swe were carried out to boost understanding: The tenses in items 1, 3, 5, 6, and 7 were altered to maintain the same tense throughout all items. Further, item 1 was changed from “little interest” to “felt less interest” and in item 8 the words “could have” were added to prevent misunderstandings in the Swedish language. This change in item 8 is in line with the English original [18] and information from Kurt Kroenke (personal communication, 2017). Finally, the last item (not included in the total score) was clarified. Table 4 contains the PHQ-8 Swe for individuals with SSc.

**Table 6:** Content validity of the Patient Health Questionnaire-8 in Swedish for individuals with systemic sclerosis

Domains [21]	Results of the analysis of interviews with quotations from health professionals and patients
Comprehensibility	The instruction, items, and response options were generally experienced as easy to understand. However, the fact that several items addressed multiple aspects was experienced to be challenging, as was estimating the number of days that each response option referred to. Some linguistic difficulties were expressed; for example concerning tense, “little interest” (Item 1) could possibly be understood as “having some interest,” while “hopeless” (Item 2) could possibly be interpreted to mean “hopeless as a person.” HPs expressed that some items could be perceived as emotionally demanding for patients, especially those with recent disease onset, and that the PHQ-8 was problem-based, not possibility-based, and could thus upset patients. Moreover, the title of the questionnaire does not clearly express what the PHQ-8 aims to assess, which may make the purpose of the questionnaire unclear. Overall, however, the PHQ-8 was experienced to be appropriate, with a suitable amount of items and response options. HPs expressed that any patient could complete PHQ-8 as long as the questionnaire is carefully introduced and responses concerning frequent symptoms of depression are followed up by HPs. Below are some experiences in quotations: “They [the items] are so concrete. I know exactly what to think about: my mental health in the last 14 days” (P7) “They [the response options] can be confusing… I don’t know what the difference is between ‘several days’ and ‘more than half of the days’” (P10) “ [difficult to understand]… this item, item 6, several questions are included in that item” (P4) “Poor appetite or eating too much [in Item 5]—what is ‘too much’?” (HP1) “Extra-demanding items, which I think can arouse patients’ emotions, like Item 2; there’s a sense of hopelessness. And Item 6: feel bad about yourself or that you’re a failure or have let yourself or your family down. I think that those [items] can be a little more emotionally demanding” (HP2)
Relevance	The time frame, the previous two weeks, was described as being appropriate, and the items were experienced as relevant and not redundant. Items 3, 4, 5, 7, 8 and the final item (not included in the total score) were described as possible referring to somatic symptoms or consequences of SSc other than depression. Here are some quotations: “I think that it’s good. It deals with daily things, and how they are, both eating and sleeping and how to feel” (P1) “No [no need to remove items]. It [PHQ-8] is also very descriptive of how you could feel” (P4) “In Item 3, to be tired is associated with the disease. You do feel [tired] almost every day; for example, I used to rest every day” (P4) “Moving slowly [in Item 8] is what we often experience at the clinic. It’s quite obvious... speaking slowly is maybe something that I do not associate… I can’t say that I noticed that patients were too slow in that way… I don’t know whether I think that it’s relevant” (HP1)
Comprehensiveness	Key symptoms of depression in SSc were described to be covered by the questionnaire. Some HPs expressed uncertainty in assessing depression, and some patients expressed limitations in personal experiences with depression. Still, items were suggested to be added, such items could cover tearfulness, meaning of life, thoughts about death or the future, demanding situations (e.g., loneliness, physical limitation, and limitations in activities), self-management strategies, and treatment adherence. Below are some quotations: “I think that they [the items] sum it up very well, everything, yes” (P9) [Suggesting adding an item] “Being diagnosed with this disease isn’t fun. If someone reads about it [the diagnosis] online, it could make them really depressed….But otherwise I think that they [the items] cover it” (HP6)

*P* patient, *HP* health professional

### Construct validity

Structural validity: The CFA for the single factor had a near “reasonable” fit with fit indicators: AIC 97.3, CFI 0.891, RMSEA 0.128, and CMIN/DF 2.47. The two-factor model provided a better fit for the data, revealing an “acceptable” fit and AIC 81.5, CFI 0.953, RMSEA 0.086, and CMIN/DF 1.66.

Hypotheses testing for construct validity: Convergent validity was supported by strong correlations between the PHQ-8 Swe and the assessment of pain (HAQ-DI VAS); fatigue (MAF); and physical role function, bodily pain, vitality, social function, and mental health (RAND-36). Moderate correlations were found between the PHQ-8 Swe and disability (HAQ-DI); gastrointestinal symptoms, lung symptoms, Raynaud’s phenomenon, digital ulcers, and overall disease severity interference with daily activities (SHAQ VAS); physical function, general health, and emotional role function (RAND-36); and disease severity of the lung system (MSS) (Table 7). Divergent validity was obtained with weak correlations between the PHQ-8 Swe and skin involvement (mRSS); disease severity of peripheral vascular, heart, and kidney systems (MSS); and disease duration (Table 7).

**Table 7:** Construct validity (correlations) of the Patient Health Questionnaire-8 in Swedish for individuals with systemic sclerosis

	PHQ-8 *r*_s_	*p* value
*Patient-reported outcome measures*^A^		
Disability, pain, and disease interference with daily activities, SHAQ		
HAQ-DI	0.63	<0.001
HAQ-DI VAS		
Pain	0.70	<0.001
SHAQ VAS		
Gastrointestinal symptoms	0.50	<0.001
Lung symptoms	0.48	<0.001
Raynaud’s phenomenon	0.41	<0.001
Digital ulcers	0.51	<0.001
Overall disease severity	0.64	
Fatigue, MAF	0.74	<0.001
Health related quality of life, RAND-36		
Physical function	− 0.50	<0.001
Physical role function	− 0.67	<0.001
Bodily pain	− 0.72	<0.001
General health	− 0.58	<0.001
Vitality	− 0.80	<0.001
Social function	− 0.76	<0.001
Emotional role function	− 0.62	<0.001
Mental health	− 0.67	<0.001
*Disease variables*^B^		
Skin involvement, mRSS	0.19	0.072
Disease severity, MSS		
Peripheral vascular system	0.20	0.061
Lung system	0.39	<0.001
Heart system	− 0.07	0.534
Kidney system	− 0.19	0.072
Disease duration^a^	0.07	0.547

The total score of PHQ-8 Swe (*n* = 89)

*r*_s_ = Spearman’s rank correlation coefficient, *SHAQ* Scleroderma Health Assessment Questionnaire, *HAQ-DI* Health Assessment Questionnaire-Disability Index, *VAS* visual analogue scale, *MAF* Multidimensional Assessment of Fatigue, *RAND-36* RAND 36-Item Health Survey, *mRSS* modified Rodnan skin score, *MSS* Medsger Severity Scale

^A^1–6 missing values occurred in certain variables

^B^1 or 2 missing values occurred in certain variables

^a^Time from the first non-Raynaud’s symptom

### Aspects of reliability

In terms of internal consistency, the Cronbach’s alpha was 0.85 and corrected item-to-total correlation had a median of 0.61 (min–max: 0.41–0.76; *n* = 87). Of the 90 patients who completed the PHQ-8 Swe on the first test occasion, 81 of them responded to it on the retest occasion. The median of the total score of the PHQ-8 Swe was 4 on the test occasion (IQR: 2–9; *n* = 89) and also 4 on the retest (IQR: 1–7; *n* = 81). The ICC was 0.83 (*n* = 81) for the total score, and the weighted kappa coefficient had a median of 0.72 for the items (min–max: 0.60–0.79). There were no significant differences between test occasions in the total score (*p* = 0.15) or in seven of the eight items (Table 4).

The total score had no floor or ceiling effects (*n* = 89).

## Discussion

This study evaluates aspects of validity and reliability of the PHQ-8 Swe for individuals with SSc. The results indicate that content validity was satisfactory overall; however, some items could be interpreted as not only related to a depressive symptom but also covering somatic symptoms related to SSc. Further, based on the interviews, some linguistic adjustments were performed. The CFA revealed a better fit for the two-factor model than the one-factor model. The PHQ-8 Swe for individuals with SSc correlates more to self-reported disability, pain, disease interference with daily activities, fatigue, and HRQL than to disease severity assessments except for a moderate association with lung disease severity. Internal consistency and the test–retest reliability of the PHQ-8 Swe total score were sufficient and there were no floor or ceiling effects.

In terms of content validity, items were expressed as generally relevant and easy to understand, though some linguistic adjustments were made to the PHQ-8 Swe to increase the understanding for individuals with SSc. Further, some HPs experienced a fear of upsetting patients due to the potentially emotionally demanding items. In general, this consideration among HPs is probably unnecessary because individuals with SSc are likely to exhibit depressive symptoms during the disease course [42], which is of important to capture.

Some items in the PHQ-8 Swe were found to cover symptoms or problems possibly attributed to the somatic symptoms of SSc. When patients (*n* = 90) completed the PHQ-8 Swe, items 1 (interest/pleasure), 3 (sleep), and 4 (tired/little energy) held the highest median scores, while items related to sleeping problems and tiredness cover symptoms that can be attributed to somatic symptoms of SSc [43]. Strong correlations were found between the PHQ-8 Swe and fatigue and vitality; others have found similar results [12]. Fatigue could be related to somatic symptoms but on the other hand, fatigue is also a part of the core criteria for depression.

Our interviews indicated that it was difficult to estimate the verbal response options in the PHQ-8. In some previous studies, the verbal response options were changed to the exact number of days, but the original verbal setting has stronger validation data [13]. Items that were suggested for inclusion in the PHQ-8 Swe involved meaning of life, demanding situations for mental health, and self-management strategies. Psychosocial support [42] and support for self-management strategies such as physical exercise are important to these patients [44]. However, the PHQ-8 consists of criteria for depression, and it would be problematic to include items beyond these criteria.

Construct validity by structural validity of the PHQ-8 Swe showed a nearly “reasonable” fit with a one-factor solution, while the two-factor model was considered to have an “acceptable” fitting model and provide a better fit to the data. The authors have not found any results in terms of structural validity regarding the PHQ-8 in patients with SSc, though the PHQ-9 previously confirmed both a single- and two-factor model without substantive differences between them [11].

Hypotheses testing for construct validity revealed weak correlations between the PHQ-8 Swe and skin involvement as well as the objectively assessed disease severity of peripheral vascular, heart, and kidney systems. This suggests divergent validity and that the PHQ-8 Swe does not capture these somatic aspects of the disease in our sample. Similar results, including physician-rated disease severity, have been described in previous studies on the PHQ-9 in SSc [12, 34]. One reason for the low correlations between the PHQ-8 Swe and disease severity could be that the disease severity of the assessed organ systems in the included sample was, at the median, mild or normal. However, the moderate association between the PHQ-8 Swe and the lung system indicates that lungs were more affected than the other assessed organ systems in our sample. Different associations between disease manifestation of the lung system and depressive symptoms have been described in SSc [4, 8], but to our knowledge, no strong associations have been presented [8]. Findings supporting convergent validity revealed moderate-to-strong correlations between the PHQ-8 Swe and disability, pain, disease interference with daily activities, fatigue, and HRQL; these results were comparable to those of previous studies on the PHQ-9 in SSc [12, 34]. There were strong correlations between the PHQ-8 Swe and pain/bodily pain, fatigue/vitality, physical role function, social function, and mental health, indicating that the PHQ-8 Swe for individuals with SSc reflects both physical and mental aspects. The medical treatment indicates that pain was a problem in our sample, which the strong correlation between pain and PHQ-8 Swe also implies, an association in agreement with previous reports [45]. Our results align with those of other studies indicating that symptoms of depression are associated with decreased HRQL more than with organ manifestations that may be life-threatening [8]. However, the relationships are complex; living with depressive symptoms can influence the person’s experienced life situation and, thus, may influence the completion of PROMs.

Although the results (*n* = 90) of the assessments of the MSS and the medical treatment may indicate severe disease for a number of patients in our sample and that patients also could have other rheumatic diseases and comorbidity, such as cardiovascular diseases, only 17% of the patients had at least moderate symptoms of depression on the PHQ-8 Swe. An earlier study of PHQ-8 in SSc has shown that 26% had at least moderate symptoms of depression which is somewhat higher than in our sample [14]. However, there are more patients in percent, in our study, with at least moderate symptoms of depression compared to the general population in Sweden and the USA [24, 46]. Nevertheless, due to the risk for overestimation when using PROMs, a diagnosis of depression must be confirmed by validated diagnostic interviews [7].

A sufficient internal consistency was found, and these results are comparable with those from the PHQ-9 in patients with SSc [11, 12]. The ICC confirmed sufficient test–retest reliability. To the best of our knowledge, the test–retest reliability of the PHQ-8 has not been assessed previously in SSc. Kroenke et al. [13] assessed the PHQ-9 for test–retest reliability in a primary care setting and found a strong association between the test occasions. The agreement between the items in our study in the test–retest was moderate to substantial [41], though a significant difference was obtained in item 3 in the test–retest procedure. The latter might be the result of fluctuations in trouble falling or staying asleep. However, the difficulties in the symptoms of depression manifesting in daily life did not differ between test occasions, implying stability in the consequences during the testing period. Thus, the test–retest reliability of the PHQ-8 Swe for individuals with SSc is satisfactory for the total score.

One limitation of our study is that convergent validity was not tested with another instrument assessing depression, such as the Center for Epidemiologic Studies Depression Scale [12]. This was not feasible because no questionnaires assessing depression have been psychometrically tested in Swedish among individuals with SSc. However, we found a strong association between the PHQ-8 Swe and mental health in RAND-36. Another limitation is that we did not evaluate the associations between the PHQ-8 Swe and all organ systems in the MSS as well as to comorbidities such as cardiovascular diseases. Nevertheless, among patients with comorbidities, there were almost equally amount that reported *no significant depressive symptoms* (PHQ-8 scores 0–4) as depressive symptoms of different severity (PHQ-8 scores 5–24) (data not shown). A further limitation is that approximately half of the patients scored no significant symptoms of depression during the latest two weeks, though they could have experienced depressive symptoms earlier [42]. On the other hand, one-third of the patients scored mild symptoms of depression, while one-sixth had moderate-to-severe symptoms of depression.

In conclusion, in this psychometric study with in majority individuals with lcSSc, the content validity was satisfactory the reliability was sufficient and there were no floor or ceiling effects. The PHQ-8 Swe was more strongly associated with self-reported disability, pain, disease interference with daily activities, fatigue, and HRQL than to disease severity assessments, except for a moderate association with lung disease severity. As health professionals struggle to support patients with SSc in self-management, identifying symptoms of depression by the PHQ-8 Swe could be one of several means. Future studies in SSc on other aspects of validity, such as investigating the PHQ-8 Swe’s ability to discriminate between patients with a confirmed diagnosis of depression by validated interviews and those without a diagnosis, are needed.

## Appendix

The diffrent steps in the psychometric evaluation of the Patient Health Questionnaire-8 in Swedish for individuals with systemic sclerosis.

**Table:**

Evaluation of content validity and linguistic adjustments of Patient Health Questionnaire-8 in Swedish (PHQ-8 Swe) for individuals with systemic sclerosis (SSc)	Evaluation of construct validity, internal consistency, test–retest reliability, and floor and ceiling effects of PHQ-8 Swe for individuals with SSc
1. First step A translation of PHQ-9 into Swedish was obtained via the PHQ Screeners [18]. Professor Kurt Kroenke approved adjustments of the Swedish translation to individuals with SSc (personal communication, 2017) A Swedish version of PHQ-8 (PHQ-8 Swe) was developed by first removing the ninth item from the PHQ-9 in Swedish	4. Last step The construct validity (structural validity) of the PHQ-8 Swe was evaluated by confirmatory factor analysis The construct validity was also evaluated in terms of convergent and divergent validity (hypotheses testing). The PHQ-8 Swe was correlated with self-reported disability, pain, disease interference with daily activities, fatigue, and health-related quality of life as well as with physician assessed skin involvement, disease severity, and disease duration Internal consistency was evaluated on the first test occasion in the test–retest procedure Test–retest reliability was assessed on patients with SSc who completed the PHQ-8 Swe on two occasions, for a mean of 11 days (*SD* 7.4) in between Floor and ceiling effects were evaluated on the first test occasion in the test–retest procedure
2. Content validity An interview guide was developed with questions about the comprehensibility, relevance, and comprehensiveness of the PHQ-8 Swe The interview guide was revised after a pilot interview with a health professional (HP) Patients (*n* = 11) completed the PHQ-8 Swe and were interviewed. HPs (*n* = 10) read and reflected on the PHQ-8 Swe based on their experiences with patients with SSc and were interviewed. The interviews were performed from September 2017 to January 2018 Interviews (MM) of patients and HPs were audio-recorded, transcribed, and analyzed with deductive content analysis The first author (MM) conducted the analysis in dialogue with the last author (CB). The text was divided into meaning units that were subsequently coded. Codes were deductively sorted with respect to domains inspired by the Consensus-Based Standards for the Selection of Health Status Measurement Instruments criteria for content validity (comprehensibility, relevance and comprehensiveness) [21]	
3. Linguistic adjustments of the PHQ-8 Swe for individuals with SSc Some linguistic adjustments of the PHQ-8 Swe were made by the research team with reference to the results of interviews. Two patient research partners reviewed and commented on the PHQ-8 Swe during this process The adjusted version was back-translated into English by a professional translator for comparison with the original. No significant changes were found. This version of the PHQ-8 Swe was further psychometric evaluated between May 2018 and January 2019 in the last step	
